# Sudden endograft collapse due to type B aortic dissection after open conversion of endovascular aortic repair

**DOI:** 10.1093/icvts/ivab333

**Published:** 2021-11-24

**Authors:** Takashi Hashimoto, Tsutomu Ito, Hideyuki Shimizu

**Affiliations:** Department of Cardiovascular Surgery, Keio University, Shinjuku, Tokyo, Japan

**Keywords:** Open conversion, Aortic dissection, Endograft collapse

## Abstract

A 58-year-old man was admitted for sudden numbness of the right leg and abdominal pain 6 months following late open conversion for endotension after endovascular aortic repair. Computed tomography demonstrated residual endograft collapse due to Stanford type B dissection. Emergent right axillobifemoral bypass was performed to perfuse the lower extremities. We performed subsequent total arch replacement with secondary thoracic endovascular aortic repair.

## INTRODUCTION

Endovascular aortic repair (EVAR) is the gold standard treatment for abdominal aortic aneurysms (AAA). Although safe, EVAR requires additional interventions in up to 30% of patients [1]. Although most endograft failures can be managed endovascularly, 2.1% require conversion to open surgery to reduce mortality. We report an extremely rare case of acute Stanford type B aortic dissection (TBAD) secondary to residual endograft collapse after conversion to open surgery.

## CASE REPORT

**Figure 1:**
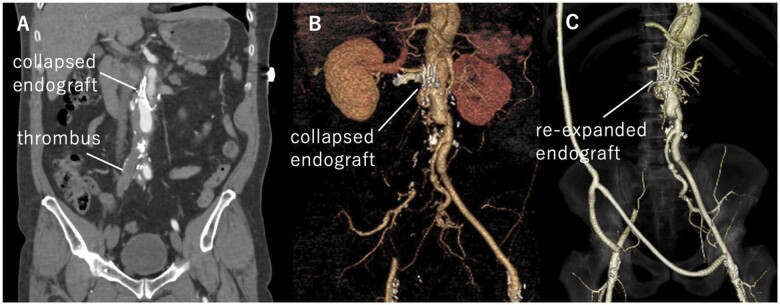
Endograft re-expansion before and after extra-anatomic bypass. Preoperative computed tomography (**A**) coronal image, (**B**) 3D image and (**C**) postoperative 3D computed tomography.

**Figure 2:**
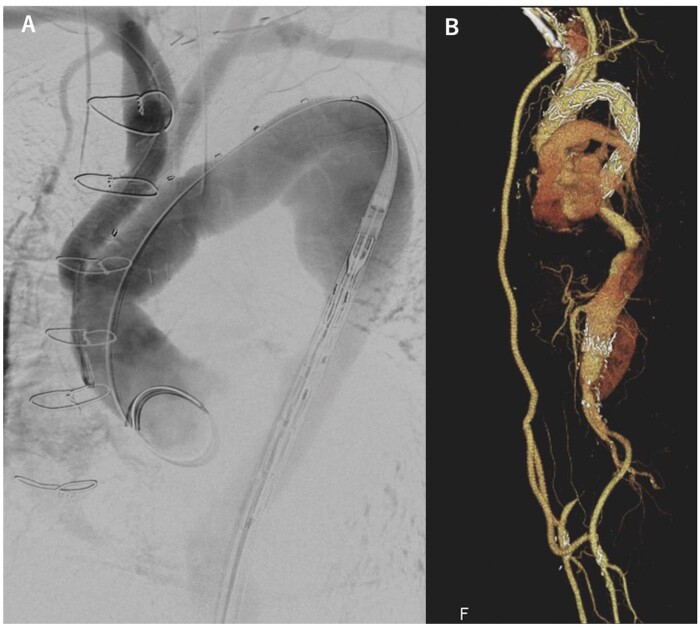
Total arch replacement with elephant trunk followed by thoracic endovascular aortic repair. (**A**) Intraoperative image of secondary thoracic endovascular aortic repair after total arch replacement with elephant trunk. (**B**) Postoperative 3D computed tomography image after thoracic endovascular aortic repair.

A 58-year-old man underwent EVAR using a 26-mm Zenith Flex AAA stent graft (Cook Medical, Bloomington, IN, USA) for a 54-mm infrarenal aortic aneurysm without an intraoperative endoleak. Regular follow-up computed tomography (CT) scans for 8 years did not demonstrate a leak. The aneurysm enlarged from 54 to 73 mm, and endotension was diagnosed. We subsequently performed open AAA repair using 20-mm InterGard Quatro graft (MAQUET, Rastatt, Baden-Württemberg, Germany), in which 2 proximal stents were preserved. Six months postoperatively, the patient complained of sudden abdominal pain and paresthaesia in the right lower extremity. Emergent CT scan demonstrated TBAD, which collapsed the residual stent graft and occluded the right leg of the Quatro graft (Fig. 1). The false lumen of the aneurysm originated from the left subclavian artery and extended to Zone 2. We performed emergent axillobifemoral bypass and completed revascularization within 5 h of limb ischaemia. No complications were observed. CT performed on postoperative day 1 demonstrated self-expansion of the residual stent graft and widening of the true lumen. To close the entry and exclude the dissecting aneurysm extending from the aortic arch to the descending aorta, we performed total arch replacement using a conventional elephant trunk procedure on POM 2 after extra-anatomic bypass (EAB), followed by thoracic endovascular aortic repair (TEVAR) on postoperative month (POM) 6 (Fig. 2).

## DISCUSSION

Twelve cases of dissection-induced endograft collapse after EVAR have been documented in the PubMed database [2–5], and some cases of leg malperfusion after open surgery have been reported. However, endograft collapse after open conversion is extremely rare and, to the best of our knowledge, it has not been reported previously. Cross-clamping of the stent graft in open conversion may lead to mild mechanical stress on the endothelium of the aorta; however, it should not have directly caused dissection in our case because the entry point was far from the endograft, i.e. at the origin of the left subclavian artery. Among 12 cases, 2 resulted in immediate mortality following rupture [4]. Of the 5 patients who underwent emergent TEVAR, 1 succumbed to non-disease-related intracranial haemorrhage [5]. Furthermore, early mortality due to the necrosis of the visceral organs occurred in 2 of the 5 patients who underwent EAB [4]. Lower-extremity amputation due to intraoperative rupture was performed in 1 patient [3]. Overall, mortality related to endograft collapse due to TBAD was as high as 42% (5/12). Although apparent results indicate that the prognosis of the TEVAR group was better than that of the EAB group, some important anatomical and technical issues underlie emergent TEVAR. We operated a dissecting arch aneurysm, which could increase the risk of retrograde Stanford type A aortic dissection during TEVAR. Previous reports have also demonstrated that passing a guidewire through a collapsed endograft is technically difficult and often needs subsequent distal thrombectomy, which is associated with delayed reperfusion [5]. However, EAB is associated with immediate reperfusion of both lower extremities by the bypass flow itself and by retrograde pressurization of the true lumen that re-expand the endograft [2]. Emergent open arch repair is also a reasonable option; however, it takes much longer to achieve reperfusion of the lower extremities.

Our study demonstrated that an additional endograft should be placed, often with thrombectomy, if ischaemia is not critical and emergent endovascular intervention is anatomically feasible, because it simultaneously achieves entry closure and revascularization. Contrastingly, if limb ischaemia is critical and requires immediate revascularization, EAB is the most efficient option, although it needs additional procedure for entry closure in the future.

**Conflict of interest:** none declared. 

## Reviewer information

Interactive CardioVascular and Thoracic Surgery thanks Davide Pacini, Hitoshi Ogino, Hiroyuki Nakajima and the other, anonymous reviewer(s) for their contribution to the peer review process of this article.

## Notes

Presented at the 11th Meeting of the German-Japanese Society for Vascular Surgery, Beppu, Japan, 7 September 2019.

## References

[1] George SG , Christos A, George AA. Lessons learned from open surgical conversion after failed previous EVAR. Ann Vasc Surg 2021;71:356–69. 32890649 10.1016/j.avsg.2020.08.122

[2] Goto Y , Hosoba S, Ogawa S, Kinoshita Y. Collapsed stent graft and severe malperfusion 2 years after endovascular aortic repair. Eur J Cardiothorac Surg 2017;52:599–600. 28605479 10.1093/ejcts/ezx195

[3] Motoji Y , Kato T, Seki J, Tsumura K, Tomita S, Okawa Y. A case of collapsed stent graft, severe lower limb ischemia, and ruptured abdominal aortic aneurysm due to type B acute aortic dissection 3 years after endovascular aneurysm repair. Ann Vasc Dis 2020;13:308–11. 33384735 10.3400/avd.cr.19-00142 PMC7751076

[4] Nomura Y , Nagao K, Hasegawa S, Kawashima M, Tsujimoto T, Izumi S et al Fatal complications of new-onset complicated type B aortic dissection after endovascular abdominal aortic aneurysm repair: report of 2 cases and literature review. Vasc Endovascular Surg 2019;53:255–8. 30572794 10.1177/1538574418819540

[5] Ostapenko A , Richard MN, Dietzek AM. Rare case of acute type B dissection causing complete collapse of EVAR stent. Vasc Endovascular Surg 2019;53:420–3. 30935297 10.1177/1538574419840873

